# Beneficial Effects of ε-Viniferin on Obesity and Related Health Alterations

**DOI:** 10.3390/nu15040928

**Published:** 2023-02-12

**Authors:** Saioa Gómez-Zorita, Iñaki Milton-Laskibar, Itziar Eseberri, Pauline Beaumont, Arnaud Courtois, Stéphanie Krisa, María P. Portillo

**Affiliations:** 1 Nutrition and Obesity Group, Department of Nutrition and Food Science, Faculty of Pharmacy and Lucio Lascaray Research Centre, University of the Basque Country (UPV/EHU), 01006 Vitoria-Gasteiz, Spain; 2 CIBERobn Physiopathology of Obesity and Nutrition, Institute of Health Carlos III, 28222 Madrid, Spain; 3 BIOARABA Health Research Institute, 01006 Vitoria-Gasteiz, Spain; 4 Univ. Bordeaux, Bordeaux INP, INRAE, OENO, UMR 1366, ISVV, F-33140 Villenave d’Ornon, France; 5 Bordeaux Sciences Agro, Bordeaux INP, INRAE, OENO, UMR 1366, ISVV, F-33170 Gradignan, France; 6 Centre Antipoison de Nouvelle Aquitaine, CHU de Bordeaux, 33076 Bordeaux, France

**Keywords:** viniferin, obesity, glucose homeostasis, dyslipidemia, hypertension, fatty liver

## Abstract

Viniferin is a phenolic compound belonging to the group of stilbenoids. In particular, ε-viniferin is a dimer of resveratrol, found in many plant genders, among which grapes (*Vitis vinifera*) are a primary source. Due to the fact that ε-viniferin is mainly present in the woody parts of plants, their use as a source of this bioactive compound is a very interesting issue in a circular economy. Both, in vitro studies carried out in pre-adipocytes and mature adipocytes and in vivo studies addressed in mice show that ε-viniferin is able to reduce fat accumulation. Moreover, it prevents the development of some obesity co-morbidities, such as type 2 diabetes, dyslipidemias, hypertension and fatty liver. ε-viniferin can be absorbed orally, but it shows a very low bioavailability. In this scenario, further research on animal models is needed to confirm the effects reported in a great number of studies; to determine which metabolites are involved, including the main one responsible for the biological effects observed and the mechanisms that justify these effects. In a further phase, human studies should be addressed in order to use ε-viniferin as a new tool for obesity management, as a nutraceutical or to be included in functional foods.

## 1. Introduction

Overweight and obesity have become a public health problem due to their high prevalence in developed and developing societies. In 2020, the estimated number of obese adults and children aged 5–19 was 764 million and 157 million, respectively. According to the latest report from the World Obesity Federation, 20% of women and 14% of men will suffer from obesity by the year 2030 [1]. Both conditions, and especially obesity, impair physical and mental health and increases the risk of developing a great number of related pathologies, such as hypertension, type 2 diabetes, cardiovascular diseases, respiratory problems and some types of cancer, among others [2]. As a result, overweight and obesity lead to high expenses for the national health systems.

Because overweight and obesity are the result of a long-term energy imbalance between calorie consumption and expenditure, the first step in the management of overweight and obesity is based on lifestyle modifications, focused on the reduction in energy intake and the increase in physical activity in order to create a negative energy balance. Nevertheless, adherence to these programs is less than desirable, especially in the long term. Moreover, pharmacotherapy is used in some cases, although the number of specific drugs approved is limited and they inevitably have negative side effects. In this scenario, the scientific community is interested in the search for bioactive compounds, naturally present in foodstuffs and plants, with anti-obesity properties, to be incorporated as complementary tools in the prevention and treatment of excessive body fat accumulation.

In this regard, different bioactive compounds (fatty acids, vitamins, pigments and phenolic compounds) are being studied [3]. Phenolic compounds are among the most frequently researched. These molecules are a large family of naturally occurring organic compounds characterized by the presence of at least one aromatic ring in its structure with one or more hydroxyl groups. Numerous in vitro studies carried out in cultured adipocytes, as well as in vivo studies addressed in animal models and human beings have shown the anti-obesity properties of a great number of these phenolic compounds. The present review focuses on the effects of ε-viniferin on obesity and some of its co-morbidities.

## 2. Chemical Structure of Viniferin

**Figure 1:**
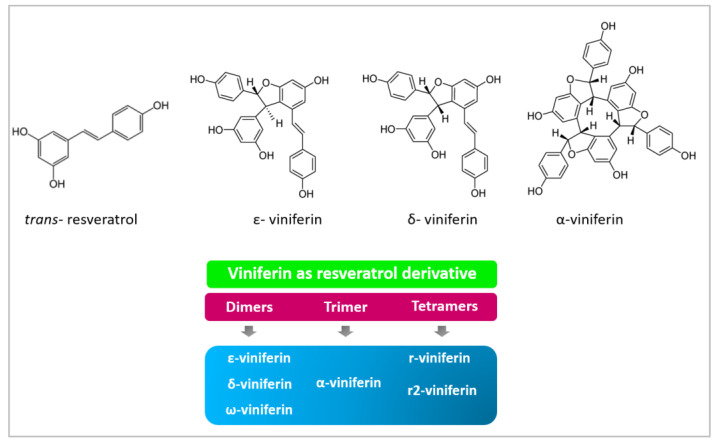
Resveratrol and viniferin chemical structures and viniferin forms classified according to the oligomer type of resveratrol [4].

Viniferin is a phenolic compound within the group of stilbenoids, also known as phytoalexins. This molecule is a derivative of resveratrol, the most researched compound in this group. Different viniferin forms are determined by the oligomerization of resveratrol as dimers, trimers or tetramers: *α*-viniferin form, a trimer of resveratrol, ε-viniferin δ-viniferin and ώ-viniferin forms, which are dimers of resveratrol, and resveratrol tetramers, such as R2-viniferin (Vitisin A) and R-viniferin (Vitisin B) (Figure 1). Moreover, the presence of two stereochemical centers in viniferin leads to the formation of four different stereoisomers [4].

ε-viniferin is probably the most studied form of viniferin, due to its many biological activities [5], such as anti-inflammatory [6], antioxidant [7], antineoplasic [8], antiobesity [9], cardioprotective [10] and neuroprotective actions [11]. As occurred with resveratrol, it was first described in grapevine leaves by Langcake and Price in 1977, who observed that it is a resveratrol dehydrodimer synthesized via oxidative cyclization [12]. In addition, it presents two stereochemical centers, at positions 7a and 8a on the dihydrofuran ring, allowing four potential stereoisomers, (±)-*trans*-ε-viniferin and (±)-cis-ε-viniferin among others. The exposition of *trans*-ε-viniferin to ultraviolet irradiation induces a rapid isomerization which leads to the formation of the cis-isomer [12]. ε-viniferin has the isomer, δ-viniferin. In 1990, the resveratrol trimer α-viniferin, which is a cyclic 42-carbon skeleton molecule, was identified as the compound responsible for the anti-inflammatory effect of a Chinese plant root (Caragana chamlagu Lamarck) [13]. To date, antioxidant and antineoplasic effects, and some beneficial outcomes against Alzheimer’s disease have also been described for this molecule [14].

## 3. ε-Viniferin Sources

ε-viniferin is present in several plant genders, such as *Vitis*, *Caragana*, *Carex*, *Hopea* and *Paonia* [12,15,16,17,18] among which grapes (*Vitis vinifera*) are a primary source. Despite ε-viniferin is present in roots, leaves, seeds, stems and barks, the woody parts of the plants are considered the main natural sources [19]. In diet, grape berries and red wine are undoubtedly the main sources. According to the Phenol Explorer database, red wine contains both δ- and ε-viniferin at 0.64 and 0.16 mg per 100 g, respectively [20].

**Figure 2:**
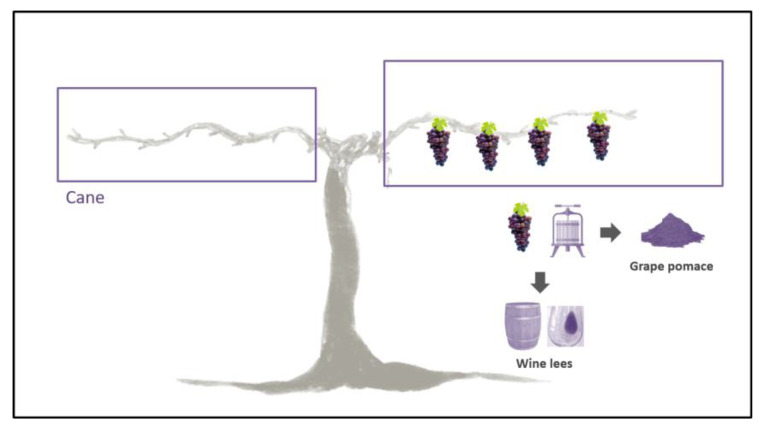
Grape canes, grape pomace and wine lees can be used for the extraction of viniferin.

Due to the fact that besides in grapes, ε-viniferin is also present in several grapevine parts, their use to extract this bioactive compound is a very interesting issue in terms of a circular economy. Effectively, in the context of sustainability, the use of by-products is a very good strategy to reduce their environmental impact by processing them to obtain an extra benefit. The wine-growing sector generates much waste derived from agricultural practices (e.g., vine shoots, leaves and stems) and the winemaking process (e.g., grape stalks, pomace and wine lees). In particular, vine shoots, also known as grapevine canes, are the most abundant waste material. Indeed, winegrowers usually select one or two grape canes that will remain on the grapevine plant to give vigor to the grapes; thus, producing great amounts of residues. The worldwide grape cane remains, generally eliminated, are between 2 and 5 tons per hectare and year [21]. These grapevine canes have a very low economic value; in fact, they are burned or incorporated into the soil to promote the degradation of organic matter and reduce the need for organic fertilizers [22]. Consequently, obtaining ε-viniferin from these residues makes them valuable, and viticulture becomes more sustainable (Figure 2) [23,24]. Apart from plant parts, further residues derived from wine production can be used to obtain ε-viniferin, grape pomace and wine lees being the most important ones (Figure 2) [25]. Grape pomace is the primary waste generated in the winemaking process, consisting of a mixture of grape skin, seeds and stems. It has been estimated that around 30–40% of phenolic compounds remain in grape pomace after winemaking.

## 4. ε-Viniferin Bioavailability

Bioavailability is a pharmacokinetic parameter that expresses the percentage of an administered compound that reaches systemic circulation and is potentially available at its target site. The bioavailability of a compound depends on its absorption and metabolism, among other factors. A recent review by El Khawand et al. (2018) [26] summarized the pharmacokinetic parameters of stilbene monomers and oligomers derived from resveratrol. It is commonly accepted that stilbenes are poorly bioavailable. However, it should be noted that there are only a few reports published so far regarding the bioavailability of resveratrol derivatives. Among these stilbenes, about ten in vivo or in vitro studies have focused on ε-viniferin. These studies are detailed in the following paragraphs.

In 2015, Willenberg et al. [27] studied the absorption of ε-viniferin using the Caco-2 cell line, a human colorectal adenocarcinoma cell line used as an in vitro model of absorption due to its similarity to the intestinal epithelium. They showed that, unlike resveratrol, ε-viniferin did not cross the intestinal cell monolayer since it was not detected in the basolateral compartment after application in the apical compartment. The authors calculated that only 16% of ε-viniferin was found in or on the cell surface. In a more recent study, Calvo-Castro et al. (2018) found a very low ε-viniferin uptake in the Caco-2 cell model. The authors also observed a very poor efflux of ε-viniferin to the basolateral compartment (less than 0.3% of the apical dose) [28].

Furthermore, Willenberg et al. (2015) [27] did not observe the metabolism of ε-viniferin in Caco-2 cells, suggesting that this dimer is not a substrate for phase II enzymes found in this intestinal cell line. In contrast, the same research group demonstrated the glucuronidation of ε-viniferin by human and rat liver microsomes. For the ε-viniferin concentration of 10 μM, 85% and 46% were glucuronidated by rat and human microsomes, respectively. Thus, although lower than resveratrol metabolism, ε-viniferin undergoes significant metabolism upon contact with liver microsomes [29]. In these studies, the metabolites of ε-viniferin were indirectly researched, either by studying the disappearance of the molecule or by using ß-glucuronidase and sulfatase, which allow the transformation of the conjugated metabolites into native molecules which are then quantified.

**Figure 3:**
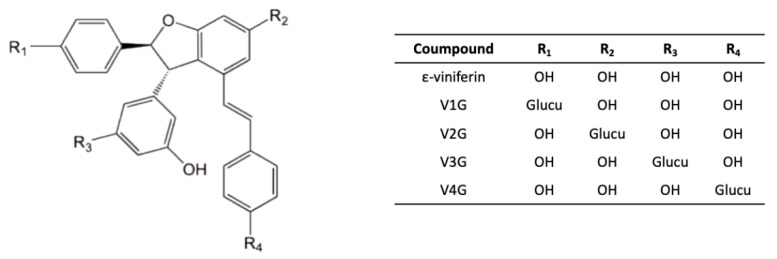
Structure of glucuronide metabolites of ε-viniferin. Glucu: glucuronide group.

Conversely, in the “Molecules of biological interest” lab in Bordeaux, we were able to quantify each of the glucuronide and sulfate metabolites of ε-viniferin, as we produced these compounds via hemisynthesis [30]. The hemisynthesis reactions revealed four glucuronidated and four sulfated isoforms, which were then separated by semi-preparative high-performance liquid chromatography. The structure of each metabolite (e.g., the position of the glucuronide group on ε-viniferin is shown in Figure 3) was then identified by proton NMR.

In this study, the enzymatic parameters of hepatic metabolism of ε-viniferin were calculated using human and rat liver fractions. Vmax/Km calculations indicated much higher glucuronidation than sulfation in rats, while both metabolic pathways contributed equally in humans. V2G glucuronide was largely predominant in rats and the four sulfated metabolites were present in small amounts, whereas in humans, two glucuronidated metabolites were predominant (V2G and V3G) and two sulfated isomers were produced. The use of liver fractions confirmed the interspecies variabilities, and in particular, the preponderance of glucuronidation in rats in contrast to humans.

These different in vitro studies have highlighted a very low passage of ε-viniferine in an intestinal barrier cellular model and an important hepatic metabolism, particularly with regard to glucuronidation. More anecdotally, an in vitro study performed on a porcine skin model showed the inability of ε-viniferin to penetrate through the skin [31].

The first in vivo study that studied the pharmacokinetic parameters of ε-viniferin was performed in mice [32]. Following oral administration, ε-viniferin (40 mg/kg body weight) rapidly reached its peak plasma concentration, after 15 min. This research group calculated the bioavailability of ε-viniferin based on the plasma concentrations measured after intravenous and oral administrations and estimated it to be 0.771%. In this study, the metabolites of ε-viniferin were not studied.

Later on, in the “Molecules of biological interest” lab in Bordeaux, an in vivo study was performed after intraperitoneal administration of 50 mg/kg body weight of ε-viniferin in rats [33]. The evolution of plasma and tissue concentrations of the native form and its metabolites were studied at different times. The work demonstrated, for the first time, the in vivo metabolism of ε-viniferin. Indeed, a strong glucuronidation, as well as a weak sulfatation, appeared one hour after ε-viniferin administration. Concerning tissue distribution, the glucuronidated forms were largely present in the liver. As for the study carried out in rat liver microsomes, V2G was found to be the major glucuronide, the four sulfated metabolites being present, which indicates a concordance between the in vitro and in vivo studies. In addition, the native form and the glucuronides were present in the kidneys. Lastly, and more surprisingly, only the native form could be quantified in brown (intra-scapular) and white (epididymal and retroperitoneal) adipose tissues, and some traces of glucuronides were accounted for in retroperitoneal adipose tissue. Caillaud et al. (2019) [34] detected traces of ε-viniferin in the brain of mice with Alzheimer’s disease after intraperitoneal administration of 10 mg/kg body weight. Nevertheless, the authors were unable to quantify it. This work suggests the passage of this compound across the blood–brain barrier.

In a recent study performed after oral administration of 20 mg/kg body weight of ε-viniferin, also addressed in the “Molecules of biological interest” lab in Bordeaux, the evolution of plasma concentrations over time and the tissue distribution at 4 h of ε-viniferin and its metabolites was researched [35]. ε-viniferin reached its Tmax 1 h after administration. The comparison of the pharmacokinetic parameters of this study with those measured by Kim et al. (2017), after the administration of twice the dose of ε-viniferin in mice, showed a good correlation between the Cmax of ε-viniferin and the administered doses [32]. We did not detect any sulfated metabolite in our study and the major metabolite found was V2G. Two plasma concentration peaks for ε-viniferin glucuronides were observed, one at 20 min and the other at 100 min (Tmax), which suggests enterohepatic circulation of these metabolites. As for plasma, glucuronides were also the major forms present in the liver, kidneys and subcutaneous adipose tissue. Moreover, for plasma, liver and kidneys, the ratios of glucuronide to ε-viniferin concentrations were much higher after oral administration than after intraperitoneal administration [33,35]. For example, in plasma, a ratio of 100 was obtained for oral administration vs. < 10 intraperitoneal administration. These results show the probable involvement of a strong intestinal metabolism in the overall metabolism of ε-viniferin. A high amount of ε-viniferin glucuronides was also observed in another study conducted in rats, in the liver and plasma, three hours after the sacrifice of the animals that were administered a daily oral dose of 252 mg/kg bw of ε-viniferin (in a *Vitis Vinifera* extract that also contains resveratrol and vitisin B), confirming the strong metabolism of this compound [36].

In adipose tissue, the concentration of ε-viniferin was always higher than in other tissues. These results suggest the storage of this stilbene in the adipose tissue, which could then represent a reservoir site in the organism. Moreover, among different adipose depots, the mesenteric adipose tissue showed the highest concentrations of ε-viniferin and glucuronides [35]. These compounds could reach this tissue via the lymphatic system without passing through systemic circulation. Indeed, the intestines and the mesentery share the same lymphatic circulation and it was shown in a study carried out in pigs that resveratrol and its glucuronide metabolites were found in the lymphatic circulation suggesting the possible passage of other stilbenes in this circulation [37].

Since ε-viniferin has a low bioavailability, it is interesting to evaluate the impact of encapsulating this dimer. In this context, encapsulation in multilamellar lipids increased the glucuronide concentrations in plasma, liver and kidney at four hours, as well as the T_1/2_, and decreased the lambda-Z (late elimination constant) of glucuronides. These results suggest a longer exposure time in the body due to slower elimination [35]. Nevertheless, it is important to take in mind that non-absorbed ε-viniferin achieves colon and can exert beneficial effects at this level. In this line, in a study carried out in intestines from rats, it was observed that the phenolic compound was able to enhance epithelial barrier function and intestinal permeability, in part modulating epithelial tight junctions and thus, these effects could contribute to the potential beneficial effects attributed to ε-viniferin in metabolic diseases [38].

Lastly, to the best of our knowledge, only one study has been carried out in humans, in which Vineatrol^®^ (a plant extract that contains, among other compounds, 75 mg of ε-viniferin) was provided to six women and six men. ε-viniferin was not detected in the plasma of these subjects (ε-viniferin was measured after hydrolysis of the metabolites by β-glucuronidase and sulfatase). This absence was certainly related to the low dose given to the subjects (about 1 mg/kg body weight) [28].

## 5. Beneficial Effects of ε-Viniferin on Obesity and Related-Diseases

The interest in ε-viniferin is based on its numerous actions, such as antioxidant, anti-inflammatory, cardioprotective, neuroprotective, anti-cancerous and anti-obesity effects, that can result beneficial for the prevention and management of a number of diseases very prevalent in our society [5]. Therefore, the potential use of this molecule to elaborate nutraceutical and functional foods is attracting great attention.

**Figure 4:**
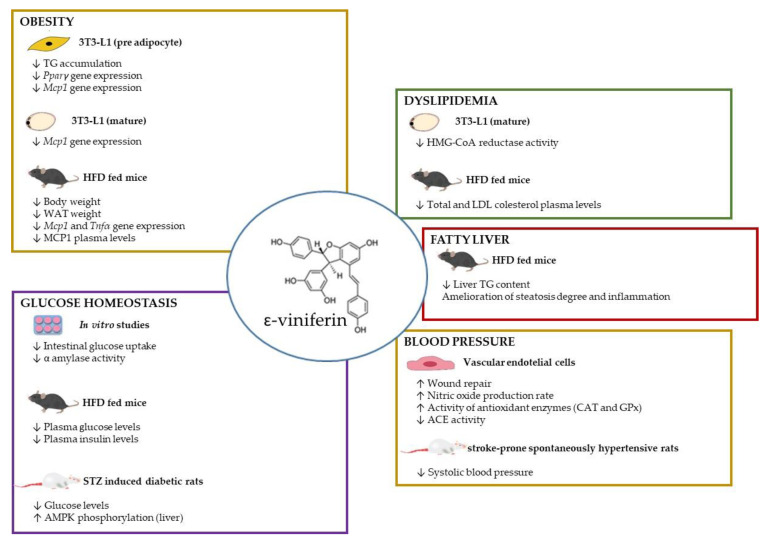
Schematic representation of ε-viniferin effects in obesity and related health alterations. ACE: angiotensin-converting enzyme, AMPK: AMP kinase, CAT: catalase, GPx: glutathione peroxidase, HFD: high-fat diet, *Mcp1*: monocyte chemoattractant protein-1, *Pparγ*: peroxisome proliferator-activated receptor gamma, TG: triglyceride, WAT: white adipose tissue.

Although the published studies demonstrating the efficacy of ε-viniferin in the management of obesity and its co-morbidities are still scarce, in the present review we are interested in gathering the reported data, obtained in in vitro and in vivo studies, to show that this is an interesting molecule that seems very promising and that further research is worth it (Figure 4).

### 5.1. Obesity

With regard to in vitro studies, Ohara et al. (2015) treated murine 3T3-L1 adipocytes during the eight days of the differentiation period with 12.5, 25 or 50 μM of ε-viniferin [39]. Interestingly, the effects of ε-viniferin were compared with those of *trans*-resveratrol. ε-viniferin, at doses of 25 and 50 μM, significantly decreased triglyceride accumulation (−37% and −72%, respectively). In contrast, only the high *trans*-resveratrol dose (50 μM) caused a significant decrease in lipid accumulation. When the gene expression of peroxisome proliferator-activated receptor gamma (*Pparγ),* the master regulator of adipogenesis, was measured, surprisingly only the highest dose of ε-viniferin reduced its expression. Due to the fact that a low-degree inflammation state in adipocytes accompanied obesity, the gene expression of the macrophage infiltration marker monocyte chemoattractant protein-1 *(Mcp1)* was also measured. Significant reductions were observed when pre-adipocytes were treated with ε-viniferin at doses of 25 and 50 μM, suggesting a decrease in the inflammatory state of the cells. Lu et al. (2017) also studied the effects of ε-viniferin on adipogeneis, although using lower doses: 2.5, 5 and 10 μM [40]. All the tested doses significantly reduced lipid accumulation in a dose-dependent manner (from 100% to 96%, 93% and 92%, respectively), indicating an inhibition of the adipogenic process.

The effects of ε-viniferin on mature adipocytes have also been analysed. Ohara et al. (2015) incubated 3T3-L1 adipocytes, on day eight of the differentiation period with 50 μM of ε-viniferin for 24 h [39]. Even though the accumulation of triglycerides was not measured at the end of the experimental period, the authors were able to evidence that *Mcp-1* mRNA level had decreased while *Pparγ* gene expression remained unchanged. These results show, as those reported by the same group in pre-adipocytes, the potential anti-inflammatory effect of ε-viniferin on adipocytes.

Regarding in vivo studies, all the reported works have been carried out in mice. The first study was carried out by Ohara et al. (2015) in five-week-old male C57BL/6J mice, which were fed a high-fat diet (60% of energy from fat) containing or not 0.2% of ε-viniferin for four weeks [39]. At the end of the experimental period, ε-viniferin treated mice showed lower body weight. This reduction was in part due to the decrease in subcutaneous, epididymal and retroperitoneal white adipose tissue weights. However, no change in mesenteric adipose depot weight was observed. In mice treated with the phenolic compound, a decrease in the mRNA levels of the inflammatory markers *Mcp-1* and tumor necrosis factor alpha (*Tnfα)* levels were observed in the epididymal adipose tissue, thus confirming the anti-inflammatory effect observed by the same group in isolated pre-adipocytes and adipocytes. ε-viniferin also reduced the gene expression and plasma levels of leptin, whereas MCP-1 plasma levels remained unchanged.

Later on, using the same animal model, mice were distributed into three experimental groups: a group fed a standard diet, a group fed a high-fat diet and a group fed a high-fat diet and treated by oral gavage with (+)-ε-viniferin at a dose of 10 mg/kg of body weight/day during the first 38 days of the experimental period, and a dose of 25 mg kg of body weight/day from day 39 to day 58. ε-viniferin treatment significantly reduced body weight compared to the non-treated animals fed with the high-fat diet. However, the weight of the animals was greater than that of the mice fed with the normal diet, meaning that the prevention of body weight gain was partial. Regarding adipose tissue weights, in sharp contrast with the study reported by Ohara et al. (2015), in which epididymal, perirenal and mesenteric adipose tissue depots were reduced, ε-viniferin only prevented the increase in the mesenteric white adipose tissue depot [40]. Unfortunately, the mechanism of action of this effect was not evaluated.

### 5.2. Glucose Homeostasis

Glucose homeostasis is the result of a complex system to maintain glucose tolerance within specific limits. When obesity is developed the high release of free fatty acids from adipose tissue induces lipotoxicity in several tissues and organs, such as the liver, skeletal muscle and pancreas, thus leading to insulin resistance and type 2 diabetes.

In this regard, different in vitro studies have been conducted lo elucidate whether the beneficial effects on glucose homeostasis attributed to ε-viniferin may be due to a decrease in intestinal glucose absorption. In order to check this hypothesis, Guschlbauer et al. (2013), studied the sodium-dependent glucose absorption in mid jejunum and ileum of Sus scofa pigs [41]. Pre-incubation of intestines with 0.2 mM of ε-viniferin reduced electrogenic intestinal glucose uptake across the epithelia. Moreover, this compound completely inhibited sodium-dependent glucose absorption into the brush border membrane vesicles (BBMV). These results indicate that ε-viniferin reduces sodium-dependent transport via sodium/glucose cotransporter 1 (SGLT1). The authors compared these effects with those of *trans*-resveratrol, and observed that whereas *trans*-resveratrol showed an inhibitory effect on glucose uptake for jejunal and ileal intestinal segments, ε-viniferin resulted in a total absence of glucose transport in ileal and jejunal BBMV, thus showing that the effect of ε-viniferin was stronger than that of *trans*-resveratrol.

In vivo studies have also been carried out addressing the effects of ε-viniferin in glucose homeostasis. In the previously mentioned study reported by Ohara et al. (2015) carried out in five-week-old male C57BL/6J mice fed a high-fat diet (60% of energy from fat) containing or not 0.2% of ε-viniferin during four weeks, plasma insulin levels were lower in treated mice despite glucose levels remained unchanged, meaning that insulin resistance was ameliorated [39]. Moreover, in the previously mentioned study reported by Lu et al. (2017), plasma glucose levels were reduced in male C57BL/6J mice fed on a high-fat diet [40].

In a recent study conducted by Liu et al. (2020), the authors examined the potential benefits of ε-viniferin administration in the prevention of type 2 diabetes and hyperlipidemia [9]. To do so, male Sprague Dawley rats were firstly fed a diet rich in fat and sucrose (chow diet containing 18% of lard and 10% of sugar) for six weeks, and then type 2 diabetes was induced by intraperitoneal injection of streptozotocin. Once type 2 diabetes onset was confirmed (fasting blood glucose level > 11.1 mmol/L), animals were fed the same high-fat high-sucrose diet, supplemented or not with ε-viniferin at doses of 30 and 60 mg/kg body weight/day, for eight additional weeks. An additional group of non-diabetic rats fed with a control diet was used as control. Both doses of ε-viniferin induced a significant reduction in basal plasma glucose level, as well as in the area under the curve in the oral glucose tolerance test, with no differences between both doses. In order to elucidate the potential involvement of AMP kinase (AMPK), a major cellular regulator of glucose metabolism recognized as a useful target for the management of type 2 diabetes, in ε-viniferin-mediated benefits on glucose homeostasis, the activation status of this kinase was studied. The Western blot analysis revealed a reduction in hepatic AMPK phosphorylation in animals fed with the high-fat high-sucrose diet, and thus a decrease in the activity of this enzyme, which was partially prevented by both doses of ε-viniferin. In fact, the immunohistochemistry analysis confirmed this result. Additionally, the potential involvement of AMPK was also studied using for this purpose molecular docking and dynamics simulation. ε-viniferin bound AMPK in the hinge region between the α- and β-unit of the kinase, a binding site also reported for other AMPK activators. Moreover, the molecular dynamics study revealed that ε-viniferin could induce a conformational change in AMPK after binding, further activating its physiological function. Altogether, the authors concluded that ε-viniferin could represent an effective preventive tool for the amelioration of metabolic disturbances such as type 2 diabetes.

The study reported by Mattio et al. (2019) also aimed at defining the mechanisms of action of ε-viniferin as an anti-diabetic molecule [42]. The authors analyzed the capacity of viniferin to inhibit pancreatic α-amylase using computational approaches in an attempt to improve the current understanding of the interaction between stilbenoids and this key protein involved in glucose metabolism. Bearing this in mind, the authors measured the enzymatic activity of porcine α-amylase in the presence or absence of racemic *trans*-ε-viniferin and *trans*-δ-viniferin. In both cases, the presence of viniferin effectively inhibited the activity of pancreatic α-amylase. When the pure enantiomers were tested, it was found that the one with the (*R,R*) configuration showed higher inhibitory capacity than the (*S,S*) configuration. According to the published results, the racemic forms of *trans*-ε-viniferin and *trans*-δ-viniferin displayed a greater inhibitory effect than their pure enantiomer forms at an equivalent total concentration. In order to better understand the results obtained in the enzymatic assay, the authors conducted a molecular docking study, which revealed that enantiomeric *trans*-viniferin forms bound α-amylase with different affinity and efficacy. Therefore, when using racemic forms (enantiomeric mixtures) a sequential binding occurs, leading to an increased inhibitory effect. Based on the results obtained, the authors concluded that viniferin effectively inhibits pancreatic α-amylase activity, especially when racemic forms of the stilbenoid are used.

### 5.3. Dyslipidemia

Dyslipidemias are frequent obesity co-morbidities. The most common ones involve increased blood triglyceride and LDL-cholesterol levels and decreased HDL-cholesterol levels.

Concerning these metabolic alterations, an in vitro study determined the effect of ε-viniferin on the activity of 3-hydroxy-3-methylglutaryl-CoA (HMG-CoA) reductase, the rate-limiting enzyme involved in cholesterol synthesis. 3T3-L1 adipocytes were incubated with 80, 120 or 160 µM of the resveratrol dimer compound and a significant dose-dependent reduction in the activity of the enzyme was observed with the three tested doses (35%, 69% and 78% inhibition, respectively) [40]. Nevertheless, it should be pointed out that the main tissue that controls serum cholesterol is not adipose tissue but liver, and thus, the analysis of the effects of ε-viniferin on the activity of hepatic HMG-CoA reductase is still needed.

In in vivo studies, Ohara et al. (2015) did not observe reductions in plasma-free fatty acids, triglycerides or total cholesterol in mice fed a high-fat diet (60% of energy from fat), containing or not 0.2% of ε-viniferin for four weeks [39]. In contrast, in the study reported by Lu et al. (2017) the two tested doses of ε-viniferin (10 and 25 mg/kg body weight/day) partially prevented the increase in total cholesterol and LDL-cholesterol plasma levels induced by the high-fat diet, although they were not able to prevent the increase in triglyceride levels [40]. Similar results were obtained by Liu et al. (2020), by using 30 and 60 mg/kg body weight/day of ε-viniferin in mice fed a diet rich in fat and sucrose [9].

### 5.4. Blood Pressure

Blood pressure is also commonly altered in obese subjects. The pathophysisological mechanisms underlying hypertension include inflammation, impairments in renin-angiotensin system, vascular dysfunction and oxidative stress among others.

Concerning this obesity co-morbidity, Zghonda et al. (2012) studied the effect of ε-viniferin on vascular endothelial cells (VEC) and renin-angiotensin system (RAS) in vitro, and on blood pressure in vivo [43].

VECs isolated from porcine pulmonary arteries were incubated with different concentrations of the phenolic compound (from 10 to 30 μM). ε-viniferin stimulated the wound repair of VECs at all the tested concentrations, when they were incubated with the molecule for 24 h, but not for eight hours. After 24 h of treatment, cell proliferation was also increased, indicating that this phenolic compound induced wound repair by increasing VEC proliferation. ε-viniferin also increased the nitric oxide production rate, in a time-dependent manner, at all the tested concentrations. Moreover, when cells were co-incubated with the phenolic compound (10 μM) and L-NAME, an inhibitor of nitric oxide synthase, ε-viniferin was not able to increase cell proliferation, meaning that nitric oxide production was implicated in the proliferative effect of ε-viniferin. Moreover, Western blot analysis revealed that at both 10 and 30 μM concentrations this molecule increased endothelial nitric oxide synthase (eNOS) phosphorylation and thus its activation.

In order to determine the potential effect of ε-viniferin on VEC protection against oxidative stress, cells were incubated with H_2_O_2_ in the presence or absence of ε-viniferin. At concentrations of 10 and 20 μM, but not at 30 μM, this molecule was able to prevent cell death and to decrease reactive oxygen species (ROS) production. In addition, it stimulated the activity of the antioxidant enzymes catalase and glutathione peroxidase (GPx), in a time-dependent manner. Thus, the decrease in intracellular ROS was probably due to the increase in antioxidant enzymes induced by the phenolic compound. The authors also observed that ε-viniferin inhibited the angiotensin-converting enzyme (ACE) activity in vitro, suggesting its potential effect in reducing blood pressure. To verify this effect, an in vivo study was carried out using fifteen-week-old male stroke-prone spontaneously hypertensive rats that were daily administered 5 mg/kg body weight of ε-viniferin by oral gavage for three weeks. The phenolic compound administration reduced systolic blood pressure but not diastolic blood pressure [43].

### 5.5. Fatty Liver

Fatty liver is characterized by an excessive fat accumulation in the liver that can be accompanied or not by inflammation and fibrosis, being its prevalence especially high in obese subjects. Regarding this alteration, two preclinical studies demonstrated the beneficial effect of ε-viniferin. In the first one, the administration of 0.2% of ε-viniferin provided in a high-fat diet was able to reduce hepatic triglyceride levels [39]. In the second study, the histological analysis revealed an amelioration in steatosis degree and inflammation in animals fed a high-fat high-sucrose diet and treated with 30 and 60 mg/kg body weight/d of ε-viniferin for 8 weeks, this effect being greater at the highest dose [9].

## 6. Conclusions and Future Research

The information included in the present review shows that, although the number of studies published so far addressing the benefits of ε-viniferin is scarce, reported data provides enough scientific evidence to state that ε-viniferin can be a promising molecule for the management of obesity and its main co-morbidities. Taking into account that vine shoots, one of the main by-products produced by the wine industry, are one of the major sources of this resveratrol dimer, the use of ε-viniferin as a bioactive molecule to improve health presents an additional benefit in terms of sustainability.

ε-viniferin can be orally absorbed in animals and it spreads to different organs, but it is poorly absorbed and undergoes a high degree of metabolism, mainly glucuronidation, thus explaining its very low bioavailability. These facts, along with the induction of significant health benefits when administered to animals suggest that its metabolites are likely to show biological activities. The higher presence of ε-viniferin and its metabolites in the adipose tissue than in other tissues could represent a therapeutic interest in the management of obesity and its related co-morbidities.

In this scenario, further research in animal models is warranted to confirm the results reported in the studies published so far, as well as to determine which metabolites are involved, including the main responsible for the biological effects observed and the mechanisms that justify these effects. In a further phase, human studies should also be addressed in order to use ε-viniferin as a new tool for obesity management, as a nutraceutical, or included in functional foods.
